# Biomaterial engineering of the tumor glycocalyx for cancer immunotherapy

**DOI:** 10.1016/j.mtbio.2026.103664

**Published:** 2026-09-10

**Authors:** Zhiyu Guo, Xu Chen, Guiye Wu, Zili Chen, Jinhong Wu, Zihao Zhou, Rongwei Xu, Meiyan Zou, Nina Li, Weiyao Feng, Xinyuan Zhao, Zhiping Wang, Li Cui

**Affiliations:** a Stomatological Hospital, School of Stomatology, Southern Medical University, Guangzhou, Guangdong, 510280, China; b School of Dentistry, University of California, Los Angeles, Los Angeles, CA, 90095, USA

**Keywords:** Tumor glycocalyx, Biomaterials, Glyco-immune interface, Tumor immunotherapy, Glycoengineering

## Abstract

The tumor glycocalyx is a dynamic biochemical and physical interface that shapes receptor accessibility, immune cell contact and glycan–lectin signaling. Cancer-associated remodeling, including hypersialylation, truncated O-glycans, altered fucosylation and mucin-rich architectures, promotes immune evasion while creating therapeutically exploitable surface features. Biomaterials offer spatial, temporal and cellular control over these distributed glycan pathways. Nanoparticles, glycopolymers, enzyme conjugates, engineered vesicles, hydrogels and synthetic cell-surface modules can suppress glycan biosynthesis, remove terminal sialic acids, dismantle mucin barriers, install bioorthogonal or antigenic cues and exploit tumor-associated glycoforms for targeting and local assembly. Related platforms can also interrupt CD24–Siglec-10/G, Siglec-15 and galectin-centered checkpoints or reprogram glycan recognition in therapeutic lymphocytes and myeloid cells, thereby enhancing trafficking, phagocytosis, antigen presentation and cytotoxicity. Translation will require control of tumor glycan heterogeneity, on-target, off-tumor activity, glycocalyx access, biodistribution and catalytic duration, together with solutions for species-specific glycan–lectin biology, enzyme immunogenicity, biomarker selection and manufacturing reproducibility. Humanized models, longitudinal glycomics, spatial glycoproteomics and modular, biomarker-guided platforms may enable conditional or reversible remodeling while minimizing unintended remodeling of physiological glycans on healthy tissues during systemic treatment, ultimately converting the tumor glycocalyx from an adaptable mechanism of immune escape into a controllable therapeutic interface.

## Highlights

• Biomaterials make the tumor glycocalyx an engineerable immunotherapy interface.
• Glycan-code engineering enables erasure, rewriting and glycoform-guided targeting.
• Glyco-immune checkpoint blockade restores phagocytic and lymphocyte responses.
• Immune-cell glycoengineering improves tumor recognition, trafficking and function.
• Translation requires selective, controllable, biomarker-guided glycocalyx remodeling.

## Graphical abstract

**Figure:**
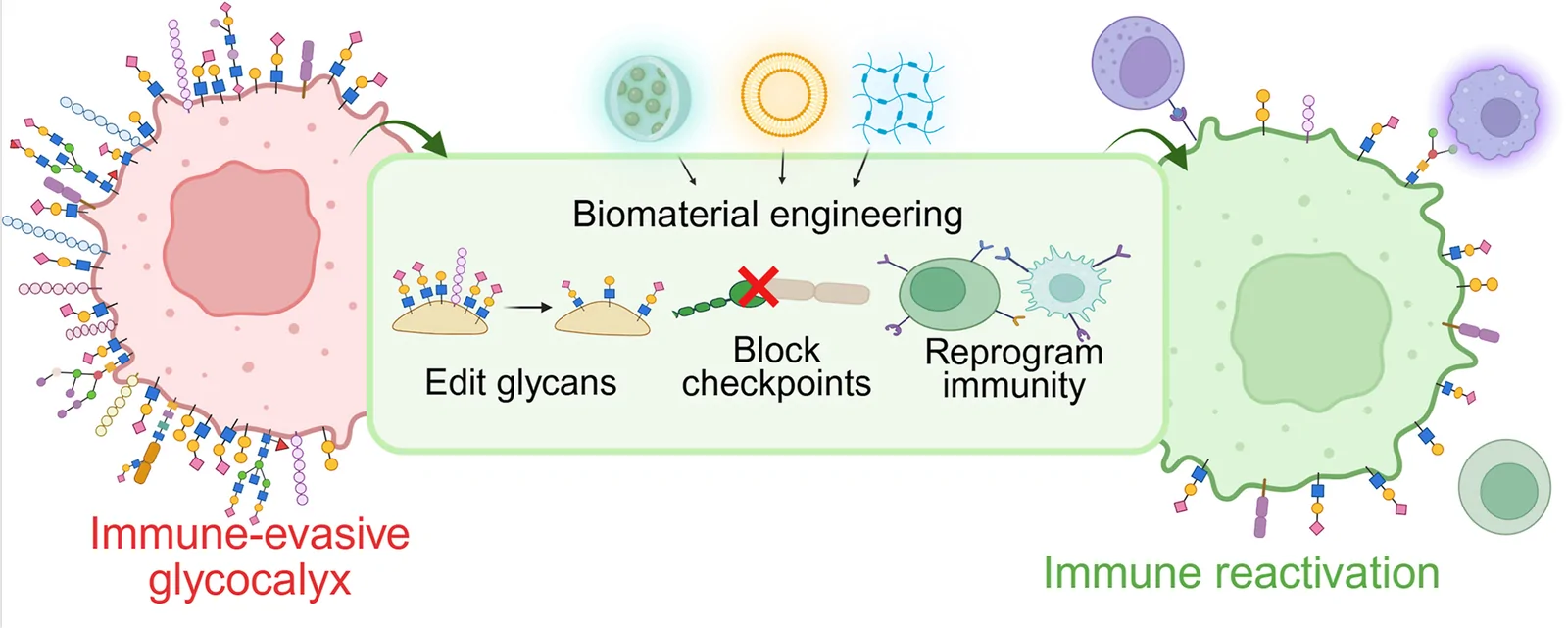

## 1 Introduction

Multicellular life depends on cells recognizing one another with sufficient accuracy to distinguish healthy neighbors from damaged cells, invading pathogens and potentially dangerous abnormalities [1]. Intercellular encounters determine whether cells adhere, migrate, exchange signals, initiate cytotoxicity or remain tolerant [2]. Such decisions begin before receptors and ligands make direct contact, because the molecular composition, spatial organization and physical properties of opposing cell surfaces already influence whether productive interactions can occur [3]. The cell surface is therefore not a smooth, passive boundary but a dynamic interface through which cells present their identity and physiological state. A major component of this interface is the glycocalyx, a carbohydrate-rich layer formed by glycans attached to proteins and lipids together with membrane-associated proteoglycans [4]. By regulating receptor accessibility, adhesion and intermembrane spacing, the glycocalyx acts not only as a protective coating but also as an information-rich layer that shapes cellular communication and recognition [5].

Malignant transformation progressively rewrites this surface information. Cancer-associated metabolic reprogramming, altered glycosylation enzymes, organelle dysfunction and changes in membrane trafficking generate hypersialylation, truncated O-glycans, aberrant fucosylation and mucin-rich glycocalyces [6]. Rather than representing isolated changes in individual glycans, these alterations collectively redefine the identity that tumor cells present to their surroundings. Expanded glycocalyces can restrict immune cell access, mask recognition sites and impair productive contact, while aberrant glycans engage Siglecs, galectins and other glycan-binding receptors that suppress phagocytic and cytotoxic responses [7,8]. Tumor-associated glycan composition, its spatial organization within the glycocalyx and its interpretation by immune cell lectins therefore converge to form a dynamic glyco-immune interface. The same glycan features that support immune escape may also distinguish malignant cells from healthy tissues, creating molecular entry points for selective therapeutic intervention [9].

**Fig. 1:**
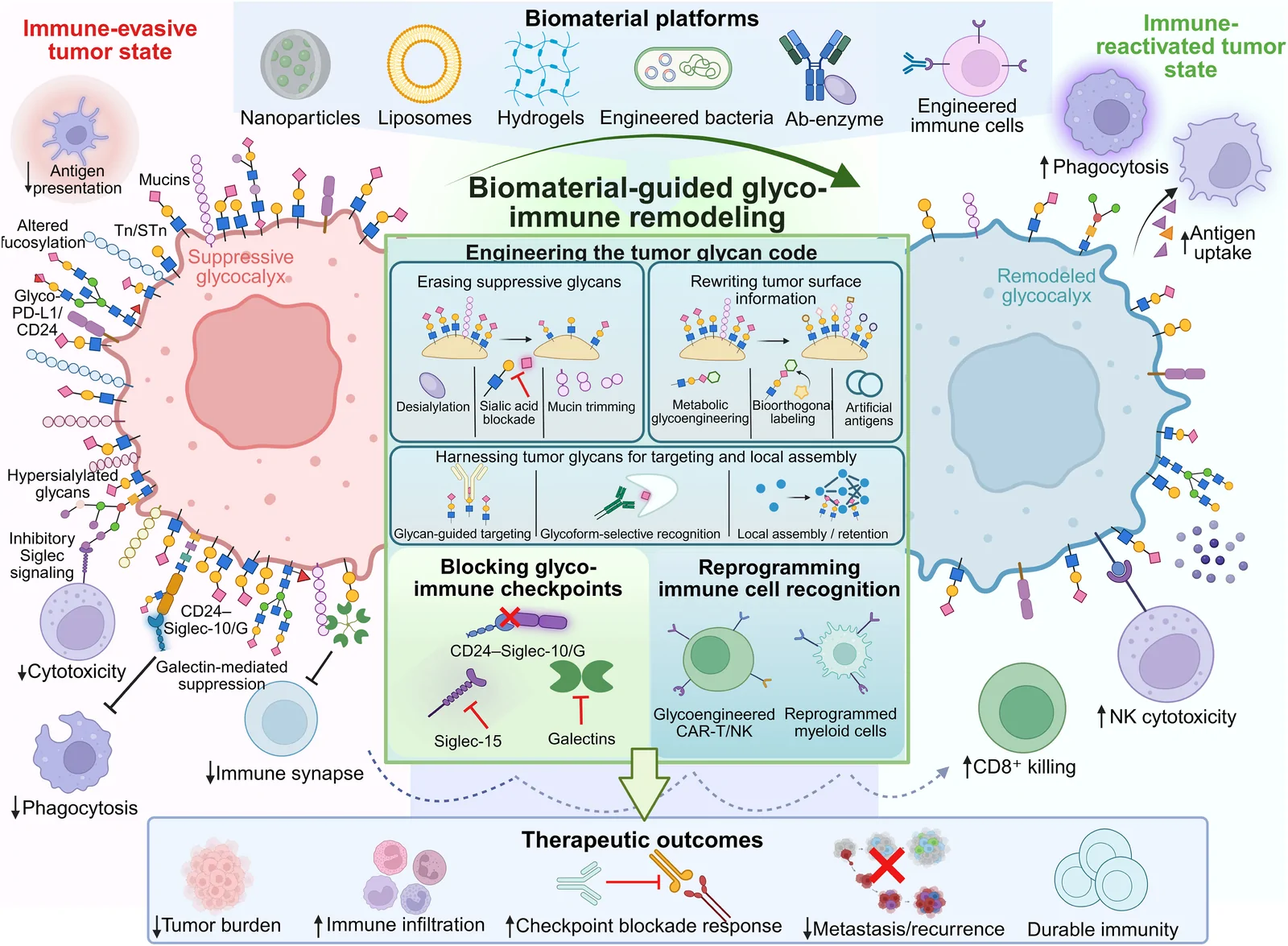
**Biomaterial engineering of the tumor glyco-immune interface for cancer immunotherapy.** Tumor-associated glycosylation establishes an immune-restrictive surface marked by hypersialylation, aberrant fucosylation, truncated Tn/STn glycans, mucin enrichment, and glycosylated checkpoint ligands, limiting antigen presentation, immune synapse formation, phagocytosis, and cytotoxicity. Biomaterial platforms, including nanoparticles, liposomes, hydrogels, engineered bacteria, antibody–enzyme conjugates, and engineered immune cells, remodel this interface through three complementary strategies: editing the tumor glycan code, blocking glyco-immune checkpoints, and reprogramming glycan recognition by immune cells. Glycan editing removes suppressive structures, installs synthetic cues, or exploits tumor-associated glycoforms for selective targeting and local assembly, whereas checkpoint blockade and immune cell engineering restore myeloid and lymphoid effector functions. Spatially controlled remodeling can also improve accessibility to tumor surface targets and strengthen productive tumor–immune cell interactions. Resulting immune reactivation enhances leukocyte infiltration, CD8^+^ T cell and NK cell activity, responsiveness to checkpoint blockade, and durable antitumor immunity, while reducing tumor burden, metastasis, and recurrence.

Precise control of the glyco-immune interface remains difficult because glycan regulation is distributed across interconnected metabolic, enzymatic and trafficking processes [10]. Biomaterials provide an engineering framework for localizing and coordinating intervention across tumor and immune cell surfaces [11]. Three therapeutic directions define this engineering framework: engineering the tumor glycan code, intercepting glyco-immune checkpoint signals and reprogramming glycan recognition and effector functions in immune cells (Fig. 1).

## 2 Tumor glycosylation and the glyco-immune interface

**Fig. 2:**
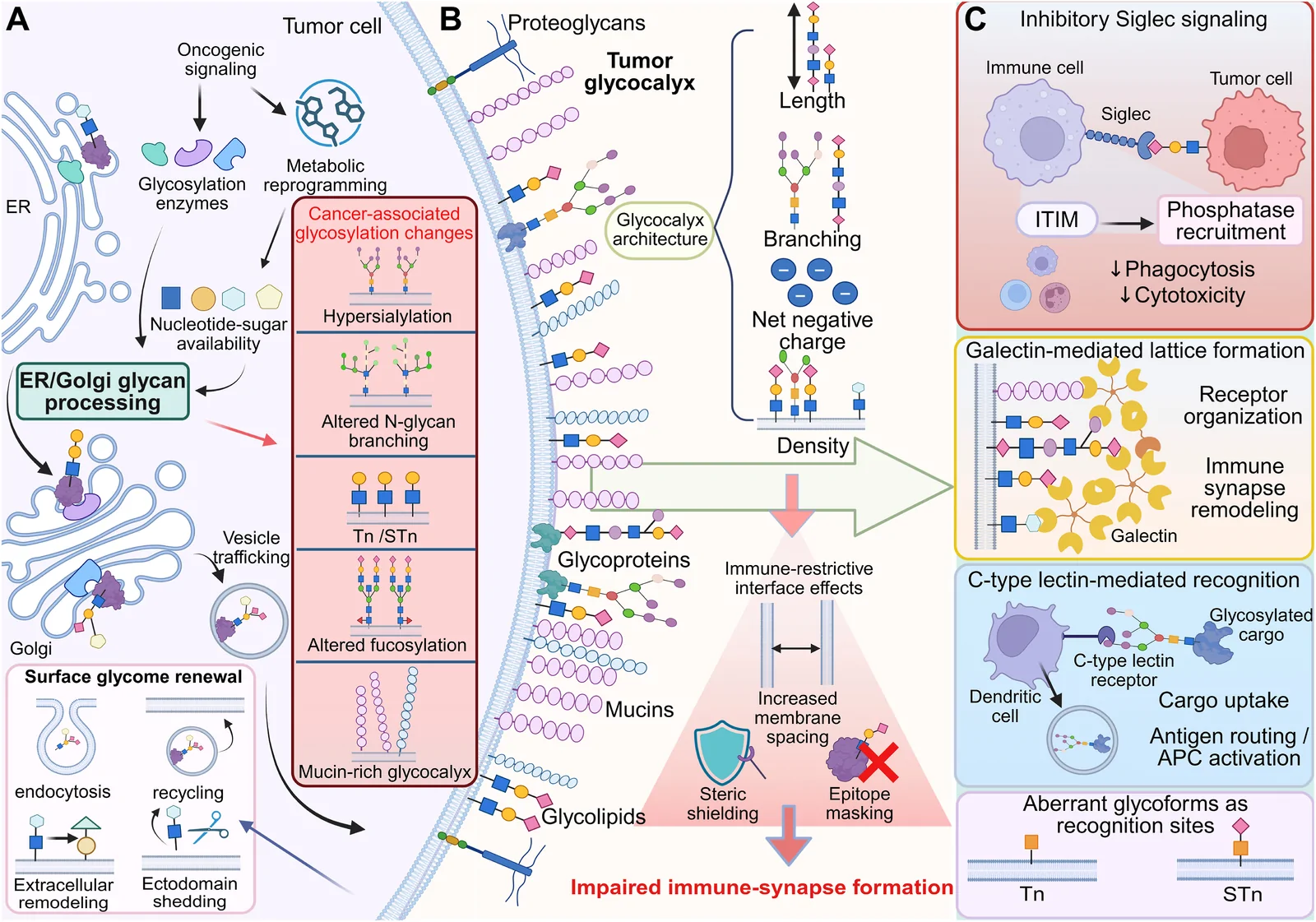
**Tumor glycosylation and immune decoding at the glyco-immune interface. (A)** Oncogenic signaling, metabolic reprogramming, and dysregulated glycosylation enzymes alter nucleotide-sugar availability and ER/Golgi glycan processing, while vesicular trafficking, endocytosis, recycling, extracellular remodeling, and ectodomain shedding continuously renew the surface glycome. Cancer-associated remodeling generates hypersialylation, altered N-glycan branching, truncated Tn/STn glycans, aberrant fucosylation, and mucin-rich glycocalyces. **(B)** Glycan composition, carrier context, and surface organization shape glycocalyx length, branching, charge, and density. Altered glycocalyx architecture increases intermembrane spacing, steric shielding, and epitope masking, thereby restricting productive immune synapse formation. **(C)** Immune cells decode tumor-associated glycans through distinct lectin systems. Inhibitory Siglecs recruit phosphatases that suppress phagocytic and cytotoxic programs, whereas galectins organize glycoprotein lattices that alter receptor distribution and immune synapse organization. C-type lectins regulate glycosylated cargo uptake, antigen routing, and activation of antigen-presenting cells. Aberrant tumor-associated glycoforms, including Tn and STn, additionally provide selective recognition sites at the tumor surface.

Cell-surface glycans are generated through compartmentalized, non-template-driven reactions in the endoplasmic reticulum and Golgi apparatus [12]. N-linked glycans are transferred to nascent proteins and subsequently trimmed, branched and capped, whereas mucin-type O-glycosylation begins with the addition of N-acetylgalactosamine and proceeds through sequential glycan extension [13]. The structures ultimately displayed at the plasma membrane are determined by nucleotide-sugar availability, the abundance and localization of glycosyltransferases and glycosidases, Golgi organization and membrane trafficking [14]. Oncogenic signaling and metabolic reprogramming alter these processes, favoring terminal hypersialylation, altered N-glycan branching, truncated Tn and sialyl-Tn O-glycans and aberrant fucosylation [15]. Endocytosis and membrane recycling further regulate the surface residence and turnover of glycosylated proteins and lipids [16] (Fig. 2A).

The biological activity of a glycan depends on how it is organized within the glycocalyx as well as on its chemical structure. Glycan length, branching, charge and density are constrained by the underlying glycoprotein, glycolipid or proteoglycan scaffold [17]. Heavily O-glycosylated mucins extend outward from the membrane and increase glycocalyx thickness, whereas changes in glycan composition and glycocalyx architecture can alter receptor mobility, clustering, retention and accessibility [18]. Dense glycan arrays also support multivalent cis and trans interactions that cannot be predicted from the affinity of an isolated glycan–lectin pair [19]. The same terminal epitope can therefore produce different cellular effects depending on its carrier protein, local copy number, nanoscale distribution and proximity to signaling receptors.

Immune cells decode this spatially organized information through Siglecs, galectins, C-type lectins and other glycan-sensitive receptors [20]. Individual glycan–lectin interactions are often weak, but multivalent presentation stabilizes receptor engagement and promotes the formation of signaling microclusters [21]. Inhibitory Siglecs can recruit phosphatases that dampen activating pathways, restrain phagocytosis or weaken lymphocyte effector functions [22]. Galectins crosslink glycoproteins into membrane lattices that modify receptor residence, immune synapse organization and myeloid cell states, whereas C-type lectins recognize defined terminal motifs and influence cargo uptake, antigen routing and antigen-presenting cell activation [23]. Glycans can also suppress immune recognition without directly initiating an inhibitory signal by masking protein epitopes, restricting receptor access or increasing the distance between opposing membranes (Fig. 2B). Conversely, tumor-associated Tn, sialyl-Tn and other aberrant glycoforms provide recognition sites that are absent or less accessible on most healthy cells [24] (Fig. 2C).

## 3 Material design principles at the glyco-immune interface

**Fig. 3:**
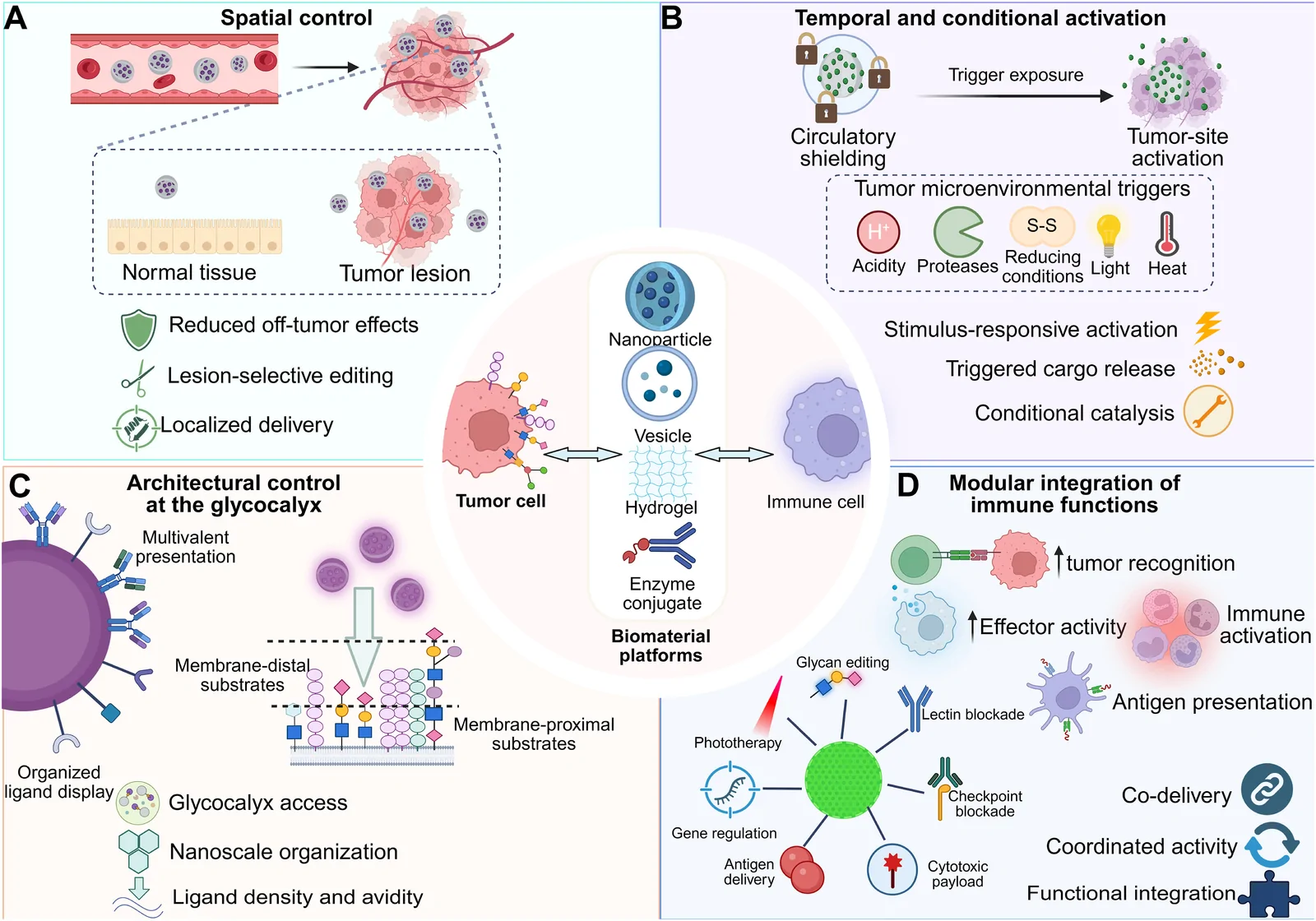
**Why biomaterials are suited to engineer the glyco-immune interface. (A)** Spatial control enables preferential accumulation and activity within tumor lesions, supporting localized glycan editing and reducing exposure of normal tissues to glycan-directed interventions. **(B)** Temporal and conditional activation can be programmed through tumor-associated acidity, proteases, reducing conditions, light, or heat, allowing stimulus-responsive unmasking, cargo release, and localized catalytic activity. **(C)** Material architecture provides control over ligand density, multivalent presentation, and nanoscale organization, thereby tuning avidity and access to membrane-distal or membrane-proximal substrates within the glycocalyx. **(D)** Modular biomaterial design integrates glycan editing with complementary therapeutic functions, including lectin or checkpoint blockade, antigen delivery, gene regulation, phototherapy, and cytotoxic payloads. Coordinated delivery across tumor and immune cell interfaces can enhance tumor recognition, effector activity, antigen presentation, and broader immune activation while maintaining spatially confined and functionally coupled intervention.

Biomaterials offer spatial, temporal and cellular control over glycan-directed intervention that conventional pharmacology rarely provides [25]. Tumor-associated glycans arise from distributed metabolic and enzymatic pathways, and many of the same structures are retained on healthy cells [26]. Systemic inhibition of glycan biosynthesis or administration of free glycosidases may therefore disrupt normal tissues, whereas unprotected nucleic acids and small molecules often show limited stability, tissue selectivity or control over exposure [27]. Engineered nanoparticles, enzyme conjugates, vesicles and hydrogels can confine glycan editing or lectin blockade to selected lesions, cell populations or membrane interfaces [28] (Fig. 3A). Activation by tumor acidity, proteases, reducing conditions, light or heat further separates systemic transport from local cargo release or catalysis, limiting glycan modification beyond the intended tissue [29] (Fig. 3B).

The glycocalyx also presents an architectural problem that materials are well suited to address. Because glycan recognition depends on ligand density, carrier context and nanoscale organization, polymers and particle surfaces can control ligand presentation to increase avidity and stabilize receptor engagement [30]. Molecular imprinting, dual-recognition designs and glycoform-selective ligands can further distinguish identical terminal glycans displayed on different carrier proteins or within distinct nanoscale environments [31]. The vertical organization of the glycocalyx creates an additional accessibility constraint, with membrane-distal glycan motifs generally more exposed than membrane-proximal substrates embedded within dense glycan and mucin layers [18]. Material properties, including surface chemistry and charge, can therefore influence access to these spatially distinct substrates and subsequent membrane binding and uptake [32] (Fig. 3C). Related principles extend to immune cell engineering, where synthetic glycan ligands, protective surface structures or glycan-binding receptors can be installed to improve tumor recognition, tissue trafficking and resistance to lectin-mediated suppression [33].

Modular assembly further allows glycan recognition and remodeling to be coupled to complementary immune functions. Glycosylation inhibition, enzymatic trimming or mucin degradation can be integrated with checkpoint blockade, cytotoxic cargo, antigen delivery, phototherapy or gene regulation rather than applied as isolated interventions [34]. Tumor-associated glycoforms may also trigger local material assembly or retention, converting aberrant surface chemistry into a spatial cue for therapeutic accumulation [35]. At the immune cell surface, synthetic modules and nucleic acid delivery systems can link glycan sensing to macrophage phagocytosis, dendritic cell antigen presentation or lymphocyte cytotoxicity [36,37] (Fig. 3D). Mechanistic classification based on the primary biological interface and initiating glycan-related action separates the principal glycan-directed event from carrier type and downstream immune outcome. Multimodal systems are therefore grouped under tumor glycan-code engineering, glyco-immune checkpoint interception or immune cell interface reprogramming according to their primary glycan-directed event, whereas cytotoxic, phototherapeutic and gene-regulatory components are treated as complementary therapeutic modules.

## 4 Engineering the tumor glycan code

Biomaterial engineering of the tumor glycan code involves three complementary operations: erasing suppressive information through biosynthetic blockade, enzymatic trimming or mucin degradation; writing chemical, glycan or antigenic cues that redirect immune recognition; and exploiting existing glycan patterns for material targeting, retention and local assembly.

### 4.1 Erasing suppressive glycan information

#### 4.1.1 Blocking tumor sialoglycan biosynthesis

Interference with sialic acid metabolism attenuates tumor hypersialylation at its source. OMV@HM-T/F is a brain-targeted, pathogen-derived outer membrane vesicle nano-interferer. Following blood–brain barrier penetration, it concurrently inhibits glycosylation precursor synthesis and sialic acid activation in glioblastoma cells, directly reducing terminal sialylation of membrane glycoproteins. The resulting glycocalyx remodeling destabilizes glycan-dependent signaling scaffolds and intercellular communication networks, thereby attenuating downstream signal amplification, glyco-immune suppression, and tumor immune evasion while restricting glioblastoma adaptability and therapeutic resistance [38]. Similarly, a core–shell nanoscale coordination polymer delivered a sialyltransferase inhibitor to tumor cells. Suppression of sialoglycan synthesis remodeled the glycocalyx and disrupted the Siglec–sialic acid checkpoint. Reduced CNT1 sialylation increased intracellular gemcitabine accumulation and amplified gemcitabine-induced immunogenic cell death. Combined NCP-STI and gemcitabine treatment elicited antitumor immunity, suppressed multiple murine tumors and reduced pulmonary metastasis [39].

#### 4.1.2 Removing terminal sialic acids from tumor glycans

Engineered antibody–sialidase bioconjugates provide a protein-based biomaterial platform for tumor-selective glycocalyx editing. Coupling a tumor-recognition module to a catalytic glycan-removal module confines desialylation to selected tumor surfaces and rewires lectin signaling at the glyco-immune interface. A trastuzumab–sialidase bioconjugate assembled through a C-terminal aldehyde tag combined HER2 recognition with localized removal of surface sialosides. Desialylation reduced inhibitory Siglec engagement on natural killer cells, increased binding to the activating receptor NKG2D, and enhanced antibody-dependent cellular cytotoxicity against tumors with moderate HER2 expression or reduced trastuzumab sensitivity [40]. In vivo, an αHER2 antibody–sialidase bioconjugate selectively stripped diverse sialoglycans from HER2-positive breast cancer cells. Localized glycocalyx editing increased immune cell infiltration and activation and prolonged survival in syngeneic breast tumor models, with therapeutic activity dependent on Siglec-E expression by tumor-infiltrating myeloid cells [41]. At the macrophage interface, a related antibody–sialidase platform removed Siglec ligands from the tumor microenvironment, halted tumor progression across several murine models, and increased responsiveness to immune checkpoint blockade. Single-cell RNA sequencing resolved macrophage-state remodeling and identified Siglec-E as the dominant receptor sensing tumor hypersialylation on tumor-associated macrophages. Genetic or therapeutic desialylation, as well as Siglec-E loss, further enhanced checkpoint blockade through macrophage repolarization [42].

Responsive non-antibody materials provide independent control over where and when catalytic desialylation occurs. A molecularly imprinted nanobiomimetic enzyme comprised a sialic acid-templated catalytic core beneath a pH-responsive inorganic–polymer shield. Tumor acidity disassembled the protective layer and exposed the hydrolytic core, enabling selective removal of tumor-associated sialic acids and disruption of inhibitory sialoglycan–Siglec signaling. Concurrent manganese ion release promoted dendritic cell activation. MINBE achieved efficient tumor desialylation in vitro and in tumor-bearing mice and markedly inhibited tumor growth [43]. In addition, aptamer-anchored microgels encapsulating neuraminidase enabled thermally controlled glyco-editing of selected tumor cells. After target cell binding, heat-triggered enzyme release locally removed surface sialic acids without genetic or metabolic manipulation. Desialylation altered lectin recognition in monocultures, cocultures and complex tumor tissue slices, relieved sialic acid-mediated immune suppression and increased natural killer cell recognition and cytotoxicity [44].

Coupling desialylation to checkpoint blockade or immune cell engagement directly remodels the tumor–immune interface. A chemically masked anti-PD-L1 nanobody–sialidase conjugate was constructed by site-specific PEGylation at a genetically encoded noncanonical amino acid. Tumor-associated MMP-2 removed the PEG mask, restoring nanobody binding and sialidase activity locally. Local activation coupled PD-L1 blockade with tumor glycocalyx desialylation, disrupting PD-1–PD-L1 and sialoglycan–Siglec signaling while improving tissue penetration. Relative to the unmasked conjugate, CAPS extended systemic half-life by 144-fold and increased tumor accumulation by 9.3-fold, enhancing immune activation and tumor control in immune checkpoint inhibitor-resistant colorectal cancer while reducing on-target, off-tumor toxicity [45]. Similarly, a bifunctional nanobody–sialidase fusion protein, Nb16-Sia, combined PD-L1 blockade with tumor glycocalyx desialylation using a sialidase derived from a human oral commensal bacterium. Concurrent targeting of PD-L1 and sialic acids disrupted coupled immune checkpoint and glyco-immune suppression. In syngeneic colorectal tumor models, Nb16-Sia outperformed monotherapies and their combination, remodeled the immune microenvironment and repolarized tumor-associated macrophages from M2-like to M1-like states through C-type lectin signaling, thereby enhancing antitumor immunity [46]. More directly, a bispecific T-cell engager (BiTE)–sialidase fusion protein combined antigen-specific T cell engagement with localized desialylation at the tumor–T cell interface. Removal of terminal sialic acids remodeled the tumor glycocalyx, enhanced immunological synapse formation and increased T cell activation and cytolysis in an antigen-dependent manner. In xenograft and syngeneic models of leukemia, melanoma and breast cancer, the fusion protein showed greater antitumor activity than the parental BiTE [47]. Moreover, tumor-targeting molecule–sialidase conjugates selectively removed sialoglycans from cancer cell surfaces, thereby dismantling the sialic acid–Siglec-5/10 glyco-immune checkpoint. Desialylation enhanced infiltration and activation of induced pluripotent stem cell-derived CAR macrophages, converting immunologically cold tumors into immune-responsive lesions. Combined glycocalyx editing and CAR-iMac transfer markedly suppressed solid tumor growth and prolonged survival in mice, while genetic deletion of Siglec-5/10 further strengthened CAR-iMac antitumor activity [48].

Integration with cytotoxic or vaccine platforms allows desialylation to generate or expose immunogenic signals in addition to relieving Siglec-mediated suppression. Local enzyme release, glycan-responsive circuits and ex vivo tumor cell editing extend this strategy from tumor destruction to antigen presentation and adaptive immune activation. *Escherichia coli* Nissle 1917 was engineered to coexpress a *Salmonella* sialidase and the photosensitizer KillerRed. After intratumoral colonization of hypoxic regions, white-light irradiation lysed the bacteria, releasing sialidase onto the tumor cell glycocalyx while activating KillerRed-derived reactive oxygen species. Removal of terminal sialic acids abolished Siglec-E engagement and protected MHC-I from lysosomal degradation, restoring antigen visibility, whereas photodynamic damage induced immunogenic cell death. In syngeneic melanoma and colorectal tumors, enhanced antigen presentation expanded cytotoxic T cells, activated NK cells, promoted M1-like macrophage polarization and generated antigen-specific memory without systemic toxicity [49]. Moreover, azido-functionalized *Escherichia coli* MG1655 was equipped with a sialic acid-responsive circuit controlling hemolysin E expression. Surface-displayed sialidase cleaved tumor cell sialoglycans, releasing free sialic acid and relieving Siglec-mediated immune suppression. The liberated metabolite activated the bacterial circuit, inducing hemolysin E production and localized tumor cell lysis [50]. Beyond these in situ approaches, an α2,3-neuraminidase-modified desialylated whole-cell ovarian tumor vaccine (DWCTV) removed terminal sialic acids and exposed Gal/GalNAc epitopes on the tumor glycocalyx.Dendritic cells pulsed with these vaccine cells acquired an MGL^bright^/Siglec-9^dim^ phenotype, increasing T cell *IFNG* expression, the CD4^+^/CD8^+^ratio, αβ T cell receptor diversity and cytotoxicity. In tumor-challenged mice, vaccination increased MGL^bright^/Siglec-E^dim^ dendritic cells in draining lymph nodes, restrained tumor growth and prolonged survival [51].

#### 4.1.3 Dismantling mucin-rich glycocalyx barriers

**Fig. 4:**
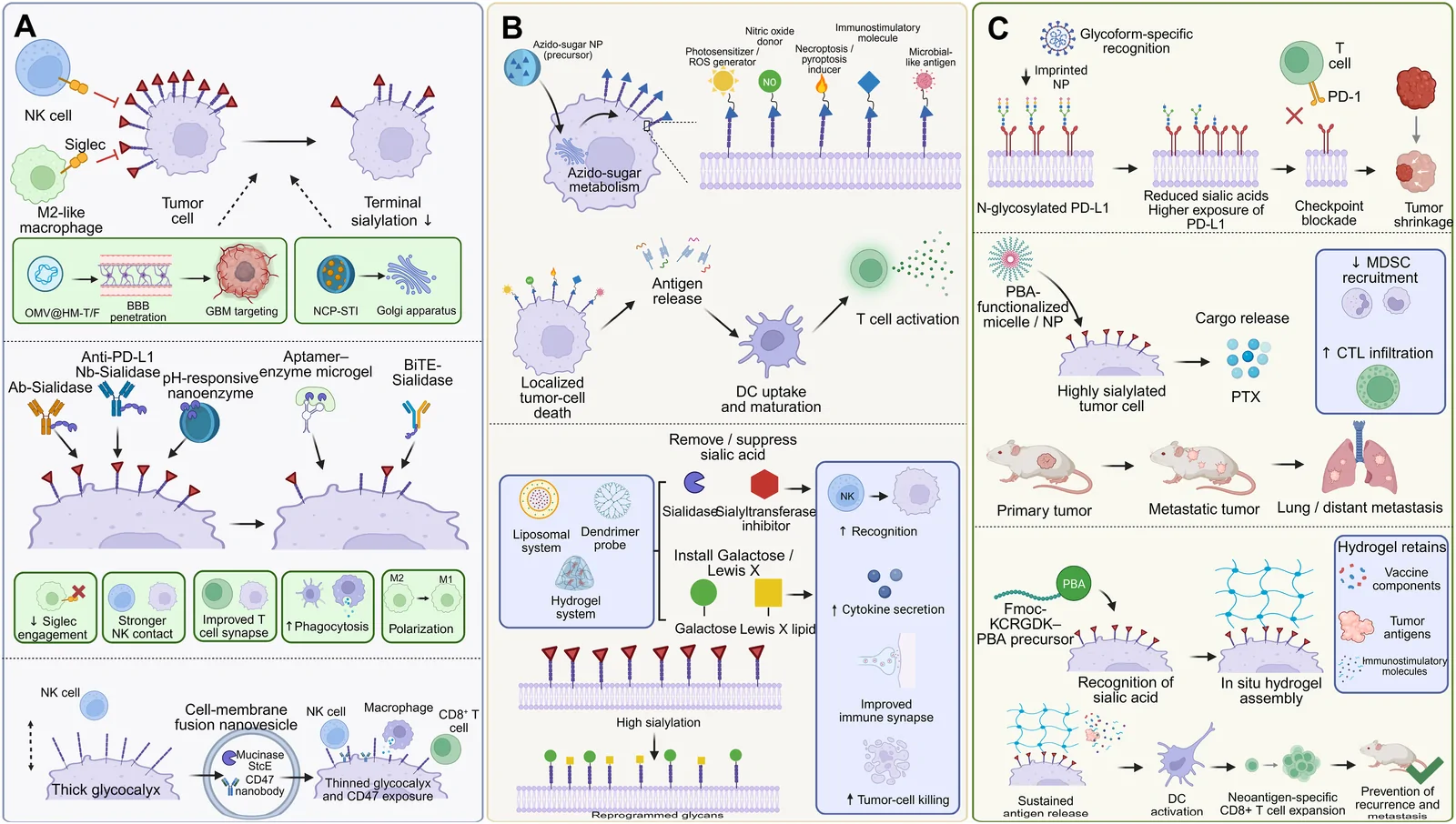
**Biomaterial engineering of the tumor glycan code. (A)** Suppressive glycan information can be erased by inhibiting sialoglycan biosynthesis, enzymatically removing terminal sialic acids, or dismantling mucin-rich glycocalyx barriers. Tumor-directed vesicles, nanomaterials, enzyme conjugates, and membrane-fusion nanovesicles reduce Siglec engagement, improve immune cell contact and synapse formation, promote phagocytosis, and favor antitumor macrophage polarization. **(B)** Tumor surface information can be rewritten through metabolic glycoengineering, bioorthogonal installation of functional molecules, or replacement of inhibitory sialic acid patterns with immunostimulatory glycan cues. Resulting surface remodeling supports antigen release and presentation, T cell activation, cytokine production, immune synapse formation, and tumor cell killing. **(C)** Pre-existing tumor glycoforms can serve as molecular addresses for selective recognition, drug delivery, and local material assembly. Glycoform-imprinted nanoparticles, sialic acid-binding carriers, and glycan-responsive hydrogels improve checkpoint accessibility, restrict suppressive myeloid recruitment, enhance cytotoxic lymphocyte infiltration, sustain local antigen delivery, and support neoantigen-specific CD8^+^ T cell responses that limit recurrence and metastasis.

The relationship between glycocalyx thickness and immune cell contact defines a physical design constraint for biomaterial and cell engineering strategies. Cancer-associated mucins and their glycosylation increased nanoscale glycocalyx thickness, creating a physical barrier that impaired cytotoxic immune cell engagement. Natural killer cell killing declined with increasing glycocalyx thickness, with changes of approximately 10 nm markedly altering tumor cell susceptibility. Engineering NK and T cells with chimeric antigen receptors partially overcame this steric defense, whereas surface display of mucinases or sialidases directly remodeled the tumor glycocalyx and further enhanced cytotoxicity [7]. More directly, cell-membrane fusion nanovesicles were engineered to co-display the mucinase StcE and a CD47 nanobody using SpyTag/SpyCatcher chemistry. Tumor-localized StcE cleaved O-glycosylated mucins, thinning the glycocalyx and increasing CD47 accessibility to the nanobody. Co-displaying StcE and the CD47 nanobody coupled physical barrier removal with checkpoint blockade, while the vesicular carrier prolonged circulation and improved tumor accumulation and biosafety relative to free StcE. In colorectal and breast tumor models, the platform increased M1-like macrophage polarization and CD8^+^ T cell infiltration, suppressing tumor growth and metastasis [52] (Fig. 4A).

### 4.2 Writing new information into the tumor glycocalyx

Writing new information into the tumor glycocalyx extends glycan editing beyond the removal of suppressive structures. Biomaterials can install bioorthogonal reaction handles, immunostimulatory carbohydrates or artificial antigens on tumor cell surfaces, thereby creating new anchoring sites, redirecting immune recognition and amplifying antitumor responses.

Bioorthogonal metabolic glycoengineering installs non-native reaction handles on tumor cells. Bioresponsive nanoassemblies delivered 1,3,4-O-acetyl-N-azidoacetylmannosamine to install azide-bearing sialic acid residues on MUC1 within the triple-negative breast cancer glycocalyx. A MUC1-lockable aggregation-induced emission theranostic reactor (AIETR), bearing dibenzocyclooctyne (DBCO) and a MUC1-binding aptamer, then engaged the installed azides and MUC1 simultaneously. Dual anchoring restricted intramolecular rotation, activating fluorescence and localized reactive oxygen species generation. Proximity-confined oxidative damage ablated membrane MUC1, triggered pyroptosis and relieved MUC1-dependent immunosuppression [53]. Similarly, triacetylated N-azidoacetyl-D-mannosamine metabolically installed azide handles on ST6Gal-1-high triple-negative breast cancer stem cells. The DBCO-functionalized liposome DLip@NO/C6 anchored to the engineered glycocalyx and co-delivered Compound 6i, a RIPK1–RIPK3–MLKL activator, with a cholesterol-based nitric oxide donor. Glycocalyx-directed delivery intensified necroptotic signaling, increased HMGB1 release and induced immunogenic necroptosis, eliminating otherwise poorly targetable cancer stem cells in vitro and in vivo [54]. More broadly, metabolic glycoengineering installed azide groups on tumor cell glycoproteins using tetraacetyl-N-azidoacetylmannosamine. Bioorthogonal ligation of DBCO–Pam3CSK4 then generated an artificial ManNAz–DBCO–Pam3CSK4 (MDP) neoantigen within the tumor glycocalyx. In syngeneic breast and lung cancer models, MDP display elicited humoral and T cell-dependent immunity, suppressed tumor growth and prolonged survival. It also sensitized poorly responsive tumors to anti-PD-1 therapy [55].

A complementary approach replaces inhibitory sialic-acid signals with stimulatory glycan cues on the tumor surface. The penta-functional dendritic probe Den@5F integrated galactose, Cy5, amino, phenylboronic acid and photo-crosslinkable mNB groups. Phenylboronic acid bound tumor-associated sialic acids, whereas light-triggered crosslinking amplified Den@5F deposition and densely displayed galactose on the tumor cell surface. Light-amplified galactose deposition masked immunosuppressive sialylation while installing immunostimulatory glycan cues, reducing immune evasion and enhancing immune cell-mediated tumor killing in vivo [56]. In addition, the triply enhanced immunotherapy drug (TEID) encapsulated galactose-conjugated streptolysin O (SLO-Gal) and neuraminidase-conjugated streptolysin O (SLO-NEU) within a hyaluronic acid shell. Tumor-associated hyaluronidase released both conjugates for streptolysin O-mediated membrane anchoring and perforation. SLO-NEU removed immunosuppressive sialic acids, whereas SLO-Gal installed stimulatory galactose cues that promoted immune cell activation and cytokine secretion. Membrane pores further facilitated cytokine entry into tumor cells, strengthening immune-mediated killing [57]. Moreover, a core–shell membrane-fusogenic liposome (MFL) was incorporated into a tumor-responsive thermosensitive hydrogel for local glycocalyx rewriting. After release and fusion with tumor cell membranes, the MFL core delivered a sialyltransferase inhibitor intracellularly to suppress sialylation, while its Lewis X-functionalized shell was incorporated into the plasma membrane. Intracellular sialyltransferase inhibition together with membrane Lewis X display replaced a sialic acid-dominated inhibitory surface with an NK-stimulatory glycan interface, enhancing tumor cell recognition and lysis by natural killer cells [58] (Fig. 4B).

### 4.3 Harnessing tumor glycans for material recognition and local assembly

Tumor-associated glycans can also function as molecular addresses for selective material recognition, lesion targeting and local therapeutic retention. Biomaterial platforms exploit defined glycoprotein glycoforms or broader sialic acid-rich glycocalyces to improve checkpoint accessibility, drug accumulation and glycan-triggered assembly at tumor sites.

Glycan-guided platforms operate across different levels of specificity, spanning defined glycoprotein glycoforms, tumor-wide sialylated surfaces and glycan-triggered material assembly. A PD-L1 N-glycan-imprinted NanoNiche was functionalized with surface-bound sialidase. Molecular imprinting enabled selective recognition of glycosylated PD-L1 on tumor cells, improving checkpoint accessibility and blockade. The conjugated enzyme simultaneously removed surface sialoglycans, relieving glycan-mediated immune suppression and promoting immune cell infiltration. NanoNiche enhanced T cell-mediated killing of PD-L1-positive breast cancer cells in vitro and improved tumor control in vivo [59]. More broadly, 3-aminophenylboronic acid-modified low-molecular-weight heparin–tocopheryl succinate micelles delivered paclitaxel to pancreatic tumors and metastases by recognizing sialic acid-rich glycocalyces. Beyond tumor targeting, the heparin-based carrier disrupted P-selectin–PSGL-1 interactions between vascular endothelial cells and myeloid-derived suppressor cells, limiting their recruitment and relieving immunosuppression. In orthotopic pancreatic cancer models, the nanoparticles accumulated in primary and metastatic lesions, remodeled the immune microenvironment and suppressed tumor growth and spontaneous metastasis [60]. Glycan recognition can also trigger local material assembly. A photodynamic therapy-primed autologous tumor cell vaccine was incorporated into an Fmoc-KCRGDK–phenylboronic acid hydrogel. Phenylboronic acid recognized sialic acid-rich tumor glycocalyces, triggering local gelation within residual surgical beds and retaining the vaccine at the tumor site. Sustained local vaccination activated neoepitope-specific CD8^+^ T cells and suppressed postoperative tumor relapse [61] (Fig. 4C).

## 5 Intercepting glyco-immune checkpoint signals

Suppressive glycan signaling is not determined by the tumor glycocalyx alone. It arises when glycosylated ligands such as CD24 engage inhibitory Siglecs or when soluble and membrane-associated galectins crosslink glycans on macrophages and lymphocytes. Blocking these communication axes uncouples glycan recognition from suppressed phagocytosis and T cell activity and can be combined with cytotoxic, myeloid-directed, or gene-editing interventions.

### 5.1 Blocking the CD24–Siglec-10/G phagocytic checkpoint

The CD24–Siglec-10/G axis transmits a glycan-dependent ‘don't-eat-me’ signal that restrains macrophage clearance of tumor cells. Biomaterial interventions disrupt this interaction directly or combine checkpoint release with tumor damage and macrophage reprogramming to extend phagocytic recovery into broader antitumor immunity.

**Fig. 5:**
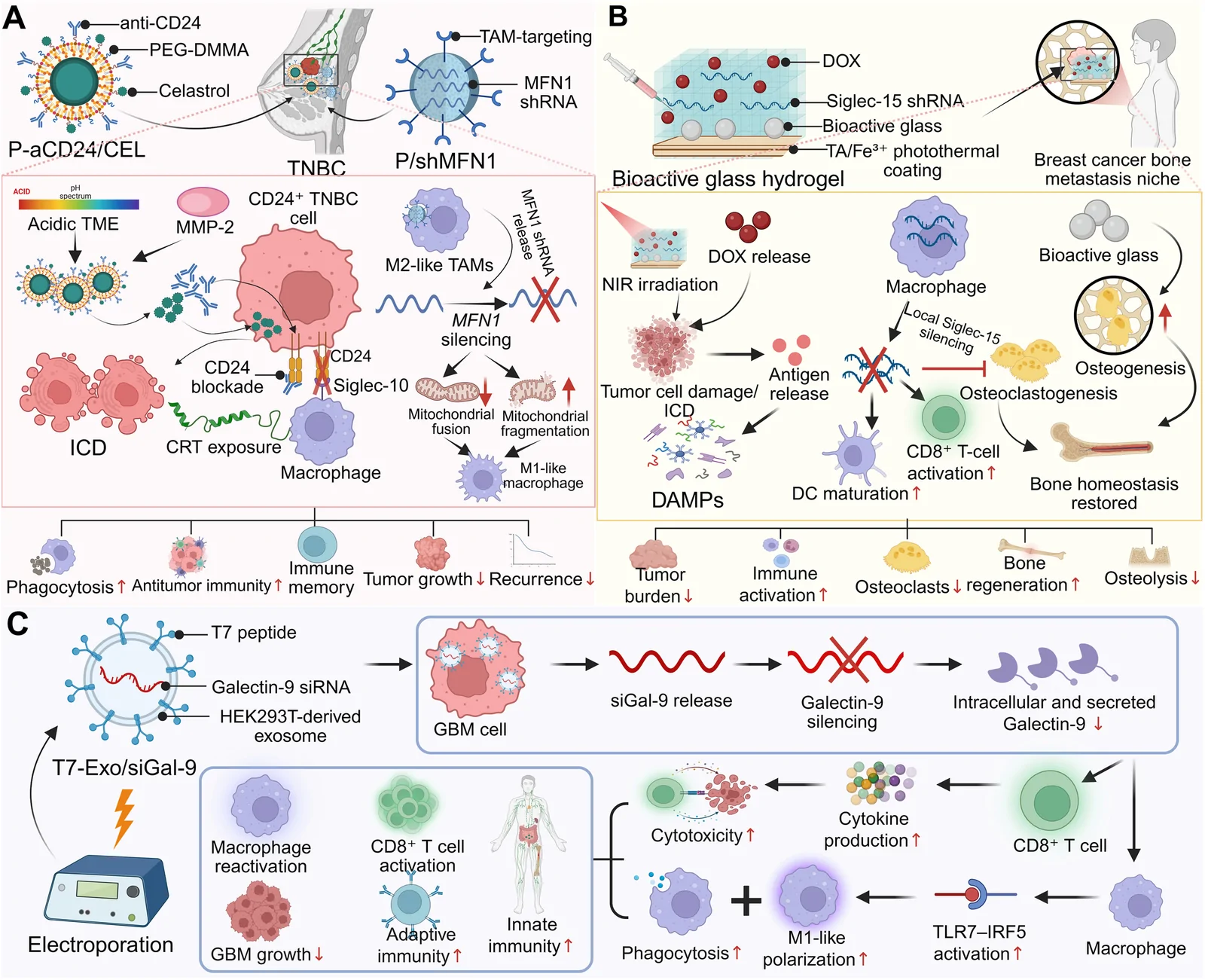
**Biomaterial interception of glyco-immune checkpoint signaling. (A)** pH- and MMP-2-responsive P-aCD24/CEL nanoparticles release anti-CD24 and celastrol within CD24^+^ triple-negative breast tumors, disrupting CD24–Siglec-10 signaling and promoting calreticulin exposure and immunogenic cell death. Concurrent delivery of *MFN1* shRNA to tumor-associated macrophages with P/sh*MFN1* suppresses mitochondrial fusion, increases mitochondrial fragmentation, and redirects M2-like tumor-associated macrophages toward an M1-like state, thereby restoring phagocytosis and strengthening antitumor immunity and immune memory while reducing tumor growth and recurrence. **(B)** An injectable bioactive-glass hydrogel carrying doxorubicin and Siglec-15 shRNA, together with a tannic acid/Fe^3+^ photothermal coating, enables NIR-triggered cargo release in breast cancer bone metastases. Tumor damage and immunogenic cell death generate antigens and DAMPs, whereas local Siglec-15 silencing supports dendritic cell maturation, CD8^+^ T cell activation, reduced osteoclastogenesis, improved osteogenesis, and restoration of bone homeostasis. **(C)** T7 peptide-engineered HEK293T-derived exosomes loaded with Galectin-9 siRNA deliver siGal-9 to glioblastoma, reducing intracellular and secreted Galectin-9 while activating TLR7–IRF5 signaling in macrophages. Reduced Galectin-9 alleviates immunosuppressive signaling, whereas macrophage reactivation promotes M1-like polarization and phagocytosis, accompanied by increased cytokine production and CD8^+^ T cell cytotoxicity.

Direct checkpoint interference provides the simplest route to restore macrophage phagocytosis. Signal-transducer peptide-anchored nanoparticles were engineered to disrupt the CD24–Siglec-10/G glyco-immune checkpoint in breast cancer without antibodies. Engagement of Siglec-expressing macrophages restored tumor cell phagocytosis and broadened downstream immune activation. Loading the nanoparticles with the CD47–SIRPα inhibitor RRx-001 enabled concurrent blockade of two myeloid checkpoints, further suppressing tumor growth with lower toxicity than the antibody-based intervention [62]. Similarly, hollow gold–iron oxide nanoparticles were functionalized with *Sambucus nigra* agglutinin. The lectin bound sialylated CD24 on melanoma cells, directly blocking the glycan-dependent CD24–Siglec-10 ‘don't-eat-me’ signal and restoring macrophage phagocytosis. Near-infrared irradiation activated the nanoparticle core to induce local photothermal damage, further increasing tumor cell engulfment in vitro and in vivo [63]. Moreover, dual nanoparticle formulations co-delivering anti-CD24 antibody/celastrol and mitofusin 1 (*MFN1*) short hairpin RNA (shRNA) coordinated tumor cell checkpoint blockade with macrophage reprogramming in triple-negative breast cancer. A pH- and MMP-2-responsive carrier released anti-CD24 and celastrol within tumors, disrupting the CD24–Siglec-10 ‘don't-eat-me’ axis, increasing calreticulin exposure and inducing immunogenic cell death to restore macrophage phagocytosis. A complementary tumor-associated macrophage-targeted nanocarrier silenced *MFN1* to remodel mitochondrial dynamics and reverse M2-like polarization. Combined treatment elicited robust antitumor immunity, durable memory and suppression of tumor progression and postoperative recurrence [64] (Fig. 5A).

### 5.2 Silencing the Siglec-15 suppressive program

Siglec-15 links glyco-immune suppression to pathological bone remodeling, making it a particularly relevant checkpoint in skeletal metastasis. Biomaterial-mediated silencing can therefore couple immune checkpoint release with tumor cell killing and osteoclast inhibition within metastatic bone lesions.

Site-directed delivery platforms have integrated Siglec-15 knockdown with chemotherapy or photothermal treatment to coordinate tumor control with preservation of bone homeostasis. An injectable bioactive-glass hydrogel co-delivered Siglec-15 shRNA and doxorubicin to breast cancer bone metastases. Photothermal heating and doxorubicin directly damaged tumor cells, while Siglec-15 silencing relieved immunosuppression and restrained osteoclastogenesis. In murine models, the hydrogel enhanced antitumor immune activation, reduced recurrence and dissemination, restored bone homeostasis and limited osteolytic destruction [65] (Fig. 5B). Similarly, a tumor-responsive hollow MnO_2_ nanoplatform was decorated with an aspartic acid octapeptide for targeting spinal metastases. It co-delivered Siglec-15 siRNA and cisplatin to lung adenocarcinoma lesions. Siglec-15 silencing in tumor cells and tumor-associated macrophages relieved glyco-immune suppression and inhibited osteoclast differentiation, while cisplatin induced tumor cell killing. In a murine spinal metastasis model, the nanoplatform accumulated at metastatic sites, suppressed tumor growth and reduced osteolytic bone destruction [66].

### 5.3 Neutralizing galectin-mediated immune suppression

Galectins sustain tumor immune escape by binding glycan ligands on cancer and immune cells, promoting T cell dysfunction, macrophage polarization and broader immunosuppressive programs. Biomaterial strategies interrupt this network by scavenging extracellular galectins, silencing their expression or editing galectin-associated checkpoints at the tumor site.

Extracellular galectins can be neutralized through multivalent glycan recognition and subsequent removal from the tumor microenvironment. A biocompatible poly(2-oxazoline) glycopolymer displayed multivalent galectin-4 ligands. Lactose-based scaffold architectures were first optimized for multivalent binding and then functionalized with a high-affinity lacto-N-tetraose-derived tetrasaccharide. The resulting glycopolymer scavenged galectin-4 from cancer cell surfaces and prevented galectin-4-induced T cell apoptosis, directly interrupting a glycan-binding pathway that supports tumor immune evasion [67]. Similarly, an antibody-mimetic polymeric nanoparticle comprised an albumin–polymer hybrid core beneath an acid-responsive PEG shell. The core displayed multivalent galactose ligands and tuftsin peptides. Tumor acidity exposed the galactose motifs, which captured intratumoral galectin-1, while tuftsin promoted macrophage-mediated clearance of the bound complexes. Galectin-1 depletion relieved glycan-binding immune suppression and restored tumor-infiltrating T cell activity [68].

Gene silencing provides a more sustained route to suppress tumor-derived galectins. Chitosan nanoparticles were loaded with galectin-1 siRNA for intranasal delivery to glioblastoma. The carrier protected the siRNA from RNase degradation and enabled rapid nose-to-brain transport and tumor cell uptake. Sequence-specific silencing reduced galectin-1 expression in murine and human glioblastoma cells and by more than 50% in tumor-bearing mice, directly suppressing a galactose-binding mediator of tumor progression and immune suppression [69]. A related intranasal formulation extended galectin-1 silencing to immune and vascular remodeling in glioblastoma. The chitosan nanoparticles reached glioblastoma through nose-to-brain transport. Galectin-1 silencing reduced myeloid suppressor cells and regulatory T cells, limited M1-to-M2 macrophage polarization and increased CD4^+^ and CD8^+^ T cell infiltration. The treatment normalized tumor vasculature and prolonged survival in tumor-bearing mice, with further survival benefits when combined with temozolomide, dendritic cell vaccination or PD-1 blockade [70].

More integrated platforms couple galectin-9 suppression to local tumor killing, innate immune reprogramming or multiplex checkpoint control. A transdermal photothermal nanosensitizer loaded with galectin-9 siRNA combined local photothermal ablation with suppression of the Gal-9–TIM-3 glyco-immune checkpoint in melanoma. Tumor cell Gal-9 silencing reduced TIM-3-mediated cytotoxic T cell exhaustion and restored effector activity, while photothermal heating induced direct tumor cell damage. The combined intervention enhanced antitumor immune responses and markedly inhibited melanoma growth [71]. Similarly, T7 peptide-decorated HEK293T-derived exosomes were electroporated with galectin-9 siRNA to enable glioblastoma-targeted glyco-immune checkpoint silencing. Exosomal delivery efficiently reduced tumor cell galectin-9 expression, relieving galectin-9-mediated immunosuppression and activating TLR7–IRF5 signaling in macrophages. The nanoformulation promoted M1-like polarization, enhanced macrophage phagocytosis and strengthened CD8^+^ T cell cytotoxicity. In vitro and in murine glioblastoma models, these coordinated innate and adaptive immune responses suppressed tumor growth and remodeled the immunosuppressive microenvironment [72] (Fig. 5C). Moreover, calcium phosphate nanoparticles co-delivering CRISPR–Cas9 ribonucleoproteins targeting PD-L1 and galectin-9 enabled simultaneous disruption of canonical and glycan-binding immune checkpoints on tumor cells. Following intratumoral administration, dual gene editing relieved PD-L1-mediated T cell suppression and Gal-9–TIM-3 signaling, while nanoparticle degradation induced Ca^2+^ overload and damage-associated molecular pattern release. The combined intervention elicited local and systemic antitumor immunity, inhibited primary and distant tumor growth, altered circulating tumor cell phenotypes through persistent dual knockout and reduced lung metastasis in mouse models [73].

## 6 Reprogramming glycan recognition and effector interfaces in immune cells

Immune cells form an engineerable side of the glyco-immune interface. Remodeling the glycocalyx of therapeutic lymphocytes, installing synthetic recognition and trafficking modules, or redirecting myeloid lectin signaling can preserve cell fitness, improve tumor engagement, and coordinate phagocytosis, antigen presentation, and cytotoxicity.

### 6.1 Rewriting the glycocalyx of effector cells to preserve function and persistence

The glycocalyx of therapeutic immune cells can determine their susceptibility to lectin-mediated suppression, differentiation state and physical engagement with tumor cells. Glycoengineering strategies can either install protective glycan structures or remove inhibitory sialoglycans, thereby preserving immune cell viability, activation and antitumor function.

**Fig. 6:**
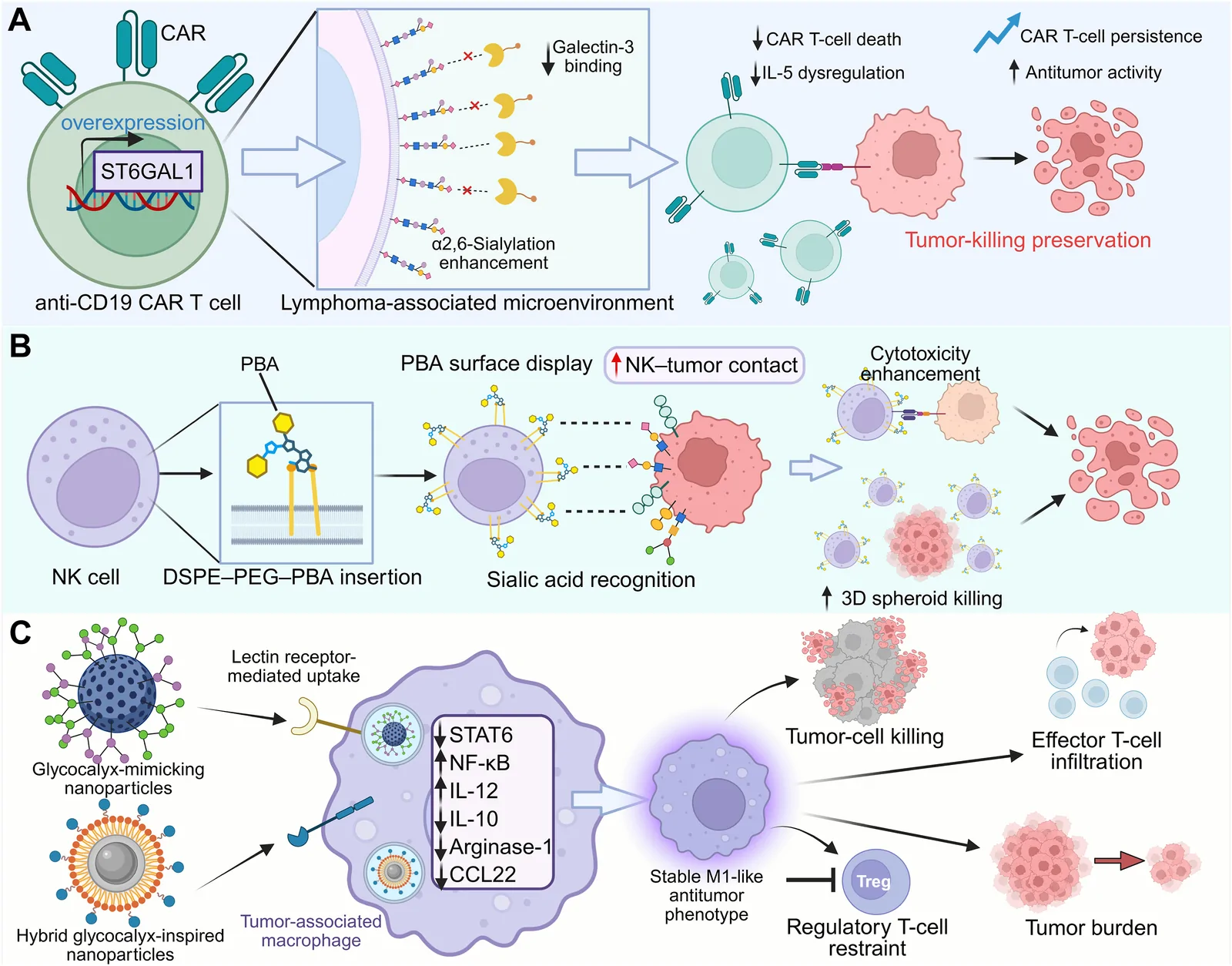
**Reprogramming glycan recognition and effector interfaces in immune cells. (A)** Glycoengineering of anti-CD19 CAR T cells by *ST6GAL1* overexpression increases surface α2,6-sialylation, limits Galectin-3 binding in the lymphoma-associated microenvironment, reduces CAR T cell death and IL-5 dysregulation, and preserves CAR T cell persistence and antitumor activity. **(B)** Membrane insertion of DSPE–PEG–PBA decorates NK cells with phenylboronic acid ligands that recognize sialic acid-rich tumor glycocalyces, strengthen NK–tumor contact, and enhance cytotoxicity, including killing of three-dimensional tumor spheroids. **(C)** Glycocalyx-mimicking nanoparticles undergo lectin receptor-mediated uptake by tumor-associated macrophages, suppressing STAT6 and activating NF-κB, with increased IL-12 and reduced IL-10, arginase-1, and CCL22. Hybrid glycocalyx-inspired nanoparticles combine surface glycan signaling with intracellular iron-oxide stimulation to stabilize a persistent M1-like antitumor phenotype and limit immunosuppressive repolarization. Macrophage reprogramming enhances tumor cell killing and effector T cell responses, while the glycocalyx-mimicking platform additionally increases effector T cell infiltration, restrains regulatory T cells, and reduces tumor burden.

Protective glycan installation and targeted desialylation address distinct forms of lectin-mediated suppression. Anti-CD19 CAR T cells were engineered to overexpress *ST6GAL1*. Increased α2,6-sialylation directly remodeled the therapeutic cell glycocalyx and reduced galectin-3 binding in lymphoma-associated microenvironments. Weakened Gal-3 engagement protected CAR T cells from Gal-3-induced death and aberrant IL-5 production without compromising tumor cell killing. In vivo, *ST6GAL1*-engineered CAR T cells showed greater persistence and stronger antitumor activity [74] (Fig. 6A). By contrast, an anti-PD-1–sialidase conjugate targeted PD-1-expressing immune cells for simultaneous checkpoint blockade and glycocalyx desialylation. Removal of cell-surface sialoglycans reduced inhibitory Siglec engagement and relieved glycan-mediated suppression of CD28 costimulation, enhancing T cell activation and cytotoxicity. In melanoma models, the conjugate reduced T cell exhaustion, promoted inflammatory macrophage polarization and improved tumor control relative to anti-PD-1 alone [75]. In a cell-intrinsic approach, CAR T cells were engineered to secrete *Clostridium perfringens* neuraminidase (CpNA). During ex vivo expansion, CpNA removed cell-surface sialic acids and restrained progressive T cell differentiation, preserving a more therapeutically competent phenotype. Neuraminidase expression also increased effector function and cytotoxicity, consistent with reduced glycocalyx-associated charge repulsion during tumor cell engagement. In leukemia, glioblastoma and melanoma models, CpNA-expressing CAR T cells achieved stronger tumor control [76].

### 6.2 Installing glycan-directed recognition and trafficking interfaces on cytotoxic lymphocytes

Therapeutic lymphocytes can be equipped with synthetic glycan-binding modules, receptor-selective glycan ligands, or glycoantigen-directed CARs to improve tumor recognition and tissue trafficking. Surface engineering can involve transient membrane insertion, chemoenzymatic glycan installation, or genetically encoded receptors.

Broad recognition of tumor-associated sialic acids can strengthen lymphocyte–tumor contact. A DSPE–PEG amphiphile bearing two phenylboronic acid (PBA) ligands was inserted into natural killer cell membranes. The displayed PBA motifs recognized sialic acid-enriched tumor glycocalyces under physiological and mildly acidic conditions, enabling glycan-directed targeting without compromising cell viability, FasL/TRAIL availability or cytokine responsiveness. PBA-decorated NK cells showed enhanced cytotoxicity against breast, liver and colorectal cancer cells and against three-dimensional breast tumor spheroids [77] (Fig. 6B). Moreover, a bifunctional glycan covalent targeting chimera comprising hydrazide-modified phenylboronic acid was used to glycoengineer T cells. The hydrazide moiety covalently coupled to oxidized galactose/N-acetylgalactosamine residues on T cell surfaces, whereas phenylboronic acid bound sialic acid-rich tumor glycocalyces, strengthening T cell adhesion and cytotoxicity. Occupation of tumor sialic acids also disrupted inhibitory sialic acid–Siglec recognition by natural killer cells, enhancing cooperative killing by T cells and NK cells [78].

Receptor-selective glycan ligands enable more selective tumor recognition and can be combined with trafficking cues. An engineered CMP-sialic acid synthetase was combined with a sialyltransferase to generate bulky, Siglec-selective sialoside ligands. One-step glycoengineering installed Siglec-2- or Siglec-3-binding ligands on NK-92MI cells, enabling glycan-directed recognition of B cell lymphoma or acute myeloid leukemia cells. The modified NK cells retained high viability while increasing granzyme release, cytokine production and tumor cell killing [79]. Moreover, high-affinity CD22 ligands were installed chemoenzymatically on NK-92MI and cytokine-induced natural killer cells. The edited glycocalyx enabled selective binding and killing of CD22-positive lymphoma cells while sparing healthy B cells. Additional display of sialyl Lewis X promoted bone marrow trafficking through E-selectin recognition, and the dual-functionalized cells suppressed B cell lymphoma in a xenograft model [80].

Glycoantigen-directed CARs enable direct recognition of tumor-associated glycoepitopes. CAR engineering can either exploit a naturally selective tumor glycoepitope or optimize glycopeptide recognition through structure-guided modification. AM52.1 is a monoclonal antibody with high specificity for sialyl-Tn (STn) and no detectable reactivity with healthy tissues. Its single-chain variable fragment (scFv) was incorporated into a second-generation CAR. The resulting CAR T cells distinguished STn-positive from STn-negative cells, eliminated STn-expressing cancer cell lines and patient-derived organoids, and controlled gastric, tubo-ovarian and colorectal mucinous peritoneal tumors in preclinical models [81]. Moreover, structure-guided engineering refined the 237 scFv, which recognizes Tn-glycopeptide epitopes generated by truncated O-glycosylation. Mutations that strengthened contacts with residues flanking the Tn glycan produced higher-affinity Tn-OTS8 binders, whereas selection against Tn-MUC1 yielded variants recognizing multiple Tn-bearing glycoproteins. Incorporated into CAR T cells, these scFvs enhanced activity against murine and human cancer cells with defective O-glycosylation [82].

### 6.3 Programming myeloid glycan sensing and antigen-presenting functions

Myeloid glycan sensing can be reprogrammed through glycocalyx-mimetic interfaces or direct manipulation of Siglec pathways. Reconfigured glycan recognition redirects macrophage polarization and phagocytosis, strengthens antigen presentation, and extends local myeloid control to T cell recruitment and immune memory.

Surface glycan presentation provides a common mechanism for macrophage repolarization, while additional intracellular stimuli or antigen cargo extend this control to adoptive cell programming and dendritic cell cross-presentation. Self-assembled glyco-nanoparticles created carbohydrate-rich surfaces that mimicked the cellular glycocalyx. When presented to primary murine peritoneal macrophages, these artificial interfaces shifted M2-like cells toward an inflammatory M1-like phenotype. Repolarization was marked by reduced CD206 and CD23, increased CD86 and coordinated changes in cytokine secretion, replacing a macrophage program associated with tumor immune suppression [83]. Similarly, glycocalyx-mimicking nanoparticles were assembled from mannose/galactose-containing diblock copolymers. Lectin receptor-mediated uptake by tumor-associated macrophages suppressed STAT6 and activated NF-κB, increasing IL-12 while reducing IL-10, arginase-1 and CCL22 and shifting macrophages toward an antitumor phenotype. Combined with anti-PD-L1 therapy, the nanoparticles reduced tumor burden, increased effector T cell infiltration and restrained regulatory T cells [84]. Moreover, hybrid glycocalyx-inspired nanoparticles combined (oligo)mannoside-functionalized glycopolymers with iron oxide. Surface glycan signaling and intracellular iron-oxide stimulation jointly stabilized a persistent M1-like macrophage phenotype, limiting immunosuppressive repolarization and increasing tumor cell killing in vitro and in vivo. Adoptive transfer of the programmed macrophages remodeled the tumor immune microenvironment and increased T cell responses [85] (Fig. 6C). In addition, a glycocalyx-mimetic nanovehicle was assembled from amphiphilic, acid-responsive glycopolymers bearing (oligo)mannosides. Neoantigen encapsulation improved antigen physicochemical properties, cellular uptake and endosomal escape, increasing cross-presentation by dendritic cells. The mannosylated nanoparticles also promoted dendritic cell maturation, cytotoxic T cell expansion and pro-inflammatory cytokine secretion [86].

Siglec-directed interventions provide a receptor-specific route to restore macrophage function, ranging from checkpoint silencing to synthetic conversion of inhibitory glycan signals. MPFS@NDs were mannose-functionalized, ultrasound-responsive nanodroplets with a perfluorohexane core. They co-delivered Siglec-G siRNA and Fe_3_O_4_ to tumor-associated macrophages. Siglec-G silencing directly relieved CD24–Siglec-G-mediated phagocytic inhibition, restoring tumor cell engulfment and antigen presentation, while Fe_3_O_4_ promoted macrophage repolarization from an M2-like to an M1-like state. Restored phagocytosis and M1-like repolarization increased T cell activation, proliferation and tumor recruitment, suppressing primary tumor growth and lung metastasis in triple-negative breast cancer models [87]. Moreover, reduction-responsive poly(disulfide amide)/lipid–PEG nanoparticles co-delivered IFN-γ and Siglec-15 siRNA to tumor-associated macrophages after systemic administration. Intracellular glutathione triggered nanoparticle disassembly and cargo release, enabling IFN-γ-driven macrophage repolarization and CXCL9 secretion to recruit T cells, while Siglec-15 silencing relieved glyco-immune suppression and restored T cell proliferation. Combined with immune checkpoint blockade, the platform increased intratumoral T cell infiltration and expansion and markedly inhibited hepatocellular carcinoma growth [88]. Beyond receptor silencing, macrophage-targeted ionizable lipid nanoparticles delivered dual circular RNAs encoding an IL-13Rα2-directed CAR and a SIGLEC9-based chimeric switch receptor. The switch receptor converted inhibitory sialoglycan–SIGLEC9 signaling into proinflammatory activation, maintaining CAR macrophages in a tumoricidal state and enhancing phagocytosis of IL-13Rα2-positive glioblastoma cells. An injectable nanoparticle–hydrogel depot around the resection cavity reprogrammed local macrophages, generated locoregional immunity and immunological memory, and suppressed postoperative tumor recurrence [89] (Table 1).

**Table 1:** Representative biomaterial strategies for engineering the tumor glyco-immune interface.

Strategy/subcategory	Primary glycan action	Biomaterial format	Key immune and therapeutic outcomes	Ref.
Sialoglycan biosynthesis blockade	Inhibition of tumor sialoglycan synthesis and disruption of sialic acid–Siglec signaling	Nanoscale coordination polymer	↑ Intracellular gemcitabine accumulation and immunogenic cell death ↓ tumor growth and lung metastasis	[39]
Tumor desialylation	Selective removal of tumor surface sialoglycans	Antibody–sialidase bioconjugate targeting HER2	↑ Immune infiltration and activation ↑ survival	[41]
Mucin barrier degradation	Cleavage of O-glycosylated mucins and increased CD47 accessibility	Nanovesicle displaying mucinase and CD47 nanobody	↑ M1 macrophage polarization and CD8^+^ T cell infiltration ↓ tumor growth and metastasis	[52]
Tumor glycocalyx rewriting	Suppression of sialylation and installation of Lewis X (Le^x^) on tumor cell membranes	Membrane fusogenic liposome in thermosensitive hydrogel	↑ NK cell recognition and tumor cell lysis	[58]
Glycan guided local assembly	Sialic acid recognition with local gelation and vaccine retention	Phenylboronic acid hydrogel	↑ Neoepitope specific CD8^+^ T cell responses ↓ postoperative recurrence	[61]
CD24–Siglec-10/G checkpoint blockade	Disruption of CD24–Siglec-10/G signaling and restoration of macrophage phagocytosis	Nanoparticle displaying signal transducer peptide	↑ Macrophage phagocytosis ↓ tumor growth	[62]
Siglec-15 silencing	Suppression of Siglec-15 signaling and osteoclastogenesis	Injectable bioactive glass hydrogel	↑ Antitumor immunity ↓ recurrence and osteolytic destruction	[65]
Galectin neutralization	Scavenging of intratumoral galectin-1 and promotion of macrophage clearance	Acid responsive polymeric nanoparticle	↑ Activity of tumor infiltrating T cells	[68]
Effector cell glycocalyx remodeling	Desialylation of PD-1^+^ immune cells and reduction of inhibitory Siglec engagement	Anti-PD-1–sialidase conjugate	↑ T cell activation and cytotoxicity ↓ T cell exhaustion and tumor growth	[75]
Lymphocyte recognition and trafficking engineering	Installation of CD22 ligands and sialyl Lewis X (sLe^x^) on NK cells	Chemoenzymatically glycoengineered NK cells	↑ Lymphoma recognition and cytotoxicity ↑ bone marrow trafficking	[80]
Myeloid glycan sensing reprogramming	Conversion of inhibitory sialoglycan–Siglec-9 signaling into proinflammatory signaling	Ionizable lipid nanoparticles for macrophage delivery with injectable hydrogel	↑ CAR macrophage phagocytosis and local antitumor immunity ↓ postoperative recurrence	[89]

## 7 Challenges and future perspectives

Tumor glycan heterogeneity and plasticity remain major barriers to reliable target selection. N-glycan branching, sialylation and other glycan features vary across cancer types, patients and metastatic sites [90,91]. Beyond this baseline heterogeneity, hypoxia, nutrient availability, inflammation and treatment can further remodel the surface glycome [92]. Glycoantigen-directed immunotherapies and molecularly imprinted materials are particularly sensitive to changes in glycan density or carrier-protein context [93,94]. Longitudinal glycomics and spatial glycoproteomics may distinguish stable targets from adaptive ones, whereas dual recognition of a glycan motif and its carrier protein may reduce escape through loss of a single glycoepitope.

Spatial selectivity is constrained by both off-tumor glycan expression and incomplete access to the tumor glycocalyx. Sialic acids, galectins and several mucins are also present in healthy tissues, raising the possibility that systemic desialylation or lectin blockade could disrupt physiological glycan-dependent functions [95]. Dense extracellular matrix and abnormal tumor perfusion can restrict the intratumoral penetration and distribution of nanomaterials and other macromolecular therapeutics [96]. Material size, charge, ligand density and circulation behavior must therefore be optimized together [97]. Local hydrogels may suit accessible or postoperative tumors, whereas disseminated disease will require systemically administered platforms activated only after tumor recognition and exposure to a local biochemical cue.

Glycan-modifying enzymes introduce distinct challenges in immunogenicity, substrate selectivity and catalytic duration. Many sialidases and mucinases used for glycocalyx editing are bacterial in origin, making immunogenicity a potential barrier to repeated administration [98]. Protein instability and proteolysis can reduce activity, whereas prolonged or poorly localized catalysis may modify neighboring healthy cells. Deimmunization and protein engineering may improve the compatibility, stability and functional selectivity of therapeutic enzymes [99]. Site-specific conjugation, cleavable shielding and conditionally activated proenzymes can further separate systemic transport from local catalysis. Pharmacodynamic analysis should identify the glycoproteins modified, the duration of glycocalyx remodeling and the extent of glycan recovery after treatment.

Preclinical interpretation remains limited by species-specific glycan–lectin biology and incomplete biomarker frameworks. Murine and human Siglecs differ in expression, ligand preference and immune cell distribution, and Siglec-E cannot represent the full repertoire of inhibitory human Siglecs [100]. Immunodeficient xenografts permit evaluation of tumor targeting or engineered cell cytotoxicity but cannot reproduce endogenous macrophage, dendritic cell and lymphocyte responses [101]. Syngeneic models preserve immune complexity yet may depend on receptor–ligand relationships that differ from those in patients. Humanized Siglec models, patient-derived organoids, tumor–immune cocultures and ex vivo tissue slices can provide complementary evidence [102]. In parallel, patient stratification may require assessment of STn abundance, linkage-specific sialylation, mucin density, carrier-protein expression and the spatial distribution of relevant Siglecs or galectins.

Manufacturing complexity may become the principal bottleneck for multimodal glyco-immune platforms. Antibody–enzyme conjugates require defined attachment sites, enzyme-to-antibody ratios and preserved binding and catalytic activities [103]. Glycopolymers and glycan-decorated nanoparticles require reproducible ligand density, molecular weight, particle size and spatial presentation because small changes in multivalency can alter avidity and cellular uptake [104,105]. Engineered immune cells add further requirements for consistent surface modification, receptor expression and functional potency. Separating recognition and effector modules may simplify optimization, quality control and regulatory assessment. The most tractable platforms may combine biomarker-guided patient selection, conditional or reversible glycocalyx editing and one defined immune intervention, such as checkpoint blockade, CAR therapy or therapeutic vaccination (Table 2).

**Table 2:** Translational challenges and design considerations for glyco-immune biomaterials.

Translational challenge	Biological and clinical basis	Material design strategy	Expected functional and translational impact	Ref.
Tumor glycan heterogeneity and target variability	Interlesional variation in glycan composition and presentation	Spatial glycome profiling and biomarker-guided target selection	↑ target consistency across lesions ↓ target mismatch	[91]
	Dependence of glycoantigen recognition on surface glycan density	Density-sensitive recognition and optimization of binding thresholds	↑ selective tumor recognition ↓ recognition of low-density physiological glycans	[93]
Spatial selectivity and tumor access	Physiological glycan expression in healthy tissues	Conditional activation and restriction of glycocalyx editing to tumor sites	↑ tumor-restricted activity ↓ activity in healthy tissues	[95]
	Restricted intratumoral distribution caused by tissue architecture and transport barriers	Optimization of particle size, surface charge, ligand density and circulation behavior	↑ intratumoral penetration and glycocalyx access ↓ nonspecific sequestration	[97]
Enzyme stability and catalytic control	Immunogenicity and limited compatibility of exogenous glycan-modifying enzymes	Compatible enzyme selection and limitation of systemic enzyme exposure	↑ tolerability during repeated administration ↓ immune responses against therapeutic enzymes	[98]
	Protein instability and insufficient control over catalytic selectivity	Protein engineering, defined conjugation and conditionally activated catalysis	↑ catalytic stability and local functional activity ↓ unintended glycocalyx remodeling	[99]
Model fidelity and patient representation	Species differences in Siglec expression, ligand preference and immune distribution	Humanized Siglec models and receptor-matched preclinical systems	↑ fidelity of glycan–lectin signaling assessment ↓ species-dependent bias	[100]
	Incomplete representation of human tumor and immune complexity in conventional models	Patient-derived organoids and tumor–immune coculture systems	↑ patient relevance of therapeutic evaluation ↑ assessment of heterogeneous responses	[102]
Manufacturing reproducibility	Variability in conjugation sites and component ratios of antibody–enzyme platforms	Site-specific conjugation and defined enzyme-to-antibody ratios	↑ product homogeneity and functional consistency ↓ batch variability	[103]
	Sensitivity of multivalent platforms to ligand density and particle surface composition	Quantitative control of ligand density and surface presentation	↑ reproducibility of avidity and cellular uptake ↓ variability in material performance	[105]

## 8 Conclusions

The tumor glycocalyx is not a passive surface coating but a dynamic biochemical and physical interface that governs receptor accessibility, lectin engagement and immune cell contact. Biomaterial strategies can intervene at complementary levels by erasing suppressive glycans, installing new chemical or antigenic cues, exploiting tumor-associated glycoforms for targeting and local assembly, blocking Siglec- and galectin-centered communication, or reprogramming glycan recognition on therapeutic immune cells. Nanoparticles, glycopolymers, enzyme conjugates, engineered vesicles, hydrogels and cell-surface modules provide spatial and temporal control that is difficult to achieve through systemic inhibition of glycan biosynthesis alone. Such control allows tumor glycosylation to function not only as a mechanism of immune escape, but also as a molecular address for localized drug delivery, catalytic editing and immune cell recruitment.

Clinical translation will require glyco-immune platforms that retain selectivity across heterogeneous tumors, achieve controllable activity at the glycocalyx and can be manufactured with defined composition and potency. Integrating patient-specific glycome profiling with modular material design may enable interventions that edit, block or reinterpret tumor-associated glycans without broadly disrupting physiological glycosylation. Aligning material architecture with the spatial organization of tumor glycans may ultimately convert an adaptable mechanism of immune escape into a controllable therapeutic interface.

## CRediT authorship contribution statement

**Zhiyu Guo:** Visualization, Writing – original draft, Writing – review & editing. **Xu Chen:** Visualization, Writing – original draft, Writing – review & editing. **Guiye Wu:** Visualization, Writing – original draft, Writing – review & editing. **Zili Chen:** Visualization, Writing – original draft, Writing – review & editing. **Jinhong Wu:** Visualization, Writing – original draft, Writing – review & editing. **Zihao Zhou:** Visualization, Writing – original draft, Writing – review & editing. **Rongwei Xu:** Visualization, Writing – original draft, Writing – review & editing. **Meiyan Zou:** Visualization, Writing – original draft, Writing – review & editing. **Nina Li:** Visualization, Writing – original draft, Writing – review & editing. **Weiyao Feng:** Visualization, Writing – original draft, Writing – review & editing. **Xinyuan Zhao:** Conceptualization, Funding acquisition, Methodology, Project administration, Supervision, Writing – original draft, Writing – review & editing. **Zhiping Wang:** Conceptualization, Methodology, Project administration, Supervision, Writing – original draft, Writing – review & editing. **Li Cui:** Funding acquisition, Methodology, Project administration, Supervision, Writing – original draft, Writing – review & editing, Conceptualization.

## Ethics approval and consent to participate

Not applicable.

## Consent for publication

Not applicable.

## Funding

This work was supported by the 10.13039/501100001809National Natural Science Foundation of China (Grant Nos. 82372905, 82573074, and 82674199), the Youth Top-notch Talent of Guangdong Special Support Program (Grant No. 0920260229), the Guangdong Provincial Science and Technology Project Foundation (Grant No. 2025A1515140097), and the Science and Technology Program of Guangzhou (Grant No. 2025A04J3464).

## Declaration of competing interest

The authors declare no competing interests.

## Data Availability

No data was used for the research described in the article.
